# Determining the effect of transforming growth factor-β1 on cdk4 and p27 in gastric cancer and cholangiocarcinoma

**DOI:** 10.3892/ol.2012.1024

**Published:** 2012-11-13

**Authors:** SUNG RYOL LEE, JAE WOOK SHIN, HYUNG OOK KIM, BYUNG HO SON, CHANG HAK YOO, JUN HO SHIN

**Affiliations:** 1 Department of Surgery, Kangbuk Samsung Hospital, Sungkyunkwan University School of Medicine, Jongno-Ku, Seoul 110-746, Republic of Korea;; 2 Department of Biology, Duke University, Durham, NC 27710, USA

**Keywords:** transforming growth factor-β1, cyclin dependent kinase 4, p27, gastric cancer, cholangiocarcinoma

## Abstract

Gastric cancer and cholangiocarcinoma are problematic throughout the world due to their destructive malignancy. In attempts to treat cholangiocarcinoma and gastric cancer, researchers often explore the effects of transforming growth factor-β1 (TGF-β1). TGF-β1 plays a crucial role in causing cell cycle arrest and fibrosis in cancer cells. The present study aimed to identify whether TGF-β1 is capable of functioning as an antitumor agent in two cancer cell lines; cholangiocarcinoma and gastric cancer. The downregulation of cyclin dependent kinase (cdk) 4 and the upregulation of p27 were investigated, in order to identify possible antitumor functions of TGF-β1. A number of different methods were implemented, including cell proliferation assay, bicinchoninic acid (BCA) assay and western blot analysis with TGF-β1, AGS (human gastric cancer cell line) and SUN-1196 (human cholangiocarcinoma cell line). In the AGS study, cdk4 values decreased from 1.000 to 0.670 and then to 0.664, with increasing TGF-β1 concentrations of 0, 0.5 and 5 ng/ml, respectively. By contrast, p27 values increased from 1.000 to 1.391 and then to 1.505, with increasing TGF-β1 concentrations of 0, 0.5 and 5 ng/ml, respectively. In the SUN-1196 study, p27 values increased from 0.548 to 0.807 and then to 0.844 with increasing TGF-β1 concentrations of 5, 25 and 50 ng/ml, respectively. Certain concentrations of TGF-β1 play antitumor roles in gastric cancer through the down-regulation of cdk4 and upregulation of p27. Certain TGF-β1 concentrations also have antitumor roles in cholangiocarcinoma through the upregulation of p27. With these results, we came a step closer to finding a cure for cholangiocarcinoma and gastric cancer.

## Introduction

Gastric cancer is one of the most common causes of cancer-related mortality and is responsible for approximately one million fatalities each year (1). Generally a fatal malignancy, gastric cancer is particularly prevalent in East Asian countries such as Korea, China and Japan (2–5). Along with gastric cancer, cholangiocarcinoma causes worldwide concern due to its destructive malignancy. Cholangiocarcinoma has a five-year survival rate of <5%. The majority of patient fatalities occur within 12 months and 60–70% of patients have a virtually inoperable condition at the time of diagnosis (6,7). In attempts to identify a cure for such cancers, researchers often explore the effects of transforming growth factor-β1 (TGF-β1). TGF-β is a pleiotropic cytokine and a versatile polypeptide. It is involved in a wide range of cellular and genetic activities, including cell proliferation, cell growth inhibition, cell morphology transformation, cell signaling pathway participation and the activation of various types of genes and proteins (8–10). Among the numerous isoforms of TGF-β (TGF-β1, TGF-β2 and TGF-β3), TGF-β1 is widely known for its inhibitory growth effect in angiogenesis and fibrogenesis (11–13). TGF-β1 inhibits the growth of nonneoplastic epithelial cells by regulating molecules related to the G1 and S phases of the cell cycle. More specifically, inhibition occurs through upregulation of mito-inhibitors including p15, p21 and p27, and through downregulation of mito-activators including cyclins and cyclin dependent kinases (cdks) (14–17). As cdks dictate cell cycle progression and p27 inhibits cdk activity by preventing the transition from G1 to S phase in the cell cycle (18), the down- and upregulation of these two factors by TGF-β1 effectively inhibits uncontrolled cell growth. Through this signaling pathway, TGF-β1 plays a crucial role in initiating cell arrest and fibrosis in cancer cells (19–22). In the present study, we aimed to identify whether TGF-β1 can function as an antitumor agent in two cancer cell lines; cholangiocarcinoma and gastric cancer. The downregulation of cdk4 and upregulation of p27 was investigated through a number of different methods including cell proliferation assay, bicinchoninic acid (BCA) assay and western blot analysis.

## Materials and methods

### Cells and culture conditions

Recombinant human TGF-β1 was provided by R&D Systems, Inc. (Minneapolis, MN, USA) and was derived from a Chinese hamster ovary cell line with the structure of a disulfide-linked homodimer. The human cholangiocarcinoma cell line (SUN-1196) and human gastric cancer cell line (AGS) were both obtained from the Korean Cell Line Bank (Seoul National University College of Medicine, Seoul, Korea). All cell lines were grown in RPMI-1640 medium (Thermo, Waltham, MA, USA) and were supplemented with glucose, 10% fetal bovine serum (FBS) and 1% penicillin/streptomycin. The cells were grown at 37°C and 5% CO_2_ in an incubator.

The study was approved by the Ethics Committee of Kangbuk Samsung Hospital, Sungkyun-kwan University School of Medicine, Seoul, Korea.

### Cell proliferation assay

Cell viability was measured by Cell Viability Reagent (Invitrogen, Grand Island, NY, USA). Cholangiocarcinoma cells (SUN-1196) and gastric cancer cells (AGS) were cultured with recombinant human TGF-β1 at concentrations of 0, 0.5, 5, 25 and 50 ng/ml for 24 h. PrestoBlue Cell Viability Reagent solution was added to each well, followed by incubation for 2 h. The cell absorbance values were measured with ELISA (Bio-Rad, Hercules, CA, USA) at a wavelength of 570 nm.

### Western blot analysis

The protein was extracted from cultured cells using PRO-PREP for Cell/Tissue Protein Extraction Solution (Intron Biotechnology, Sungnam, Korea) and protein concentration was determined by BCA Protein Assay kit (Thermo). For western blot analysis, protein samples (20 *μ*g) were subjected to sodium dodecyl sulphate polyacrylamide gel electrophoresis (SDS-PAGE) and then transferred to polyvinylidene difluoride (PVDF) membranes. The membranes were incubated with primary antibodies to cdk4 (34 kDa, 1:2000, rabbit polyclonal; Abcam, Cambridge, UK), p27 (27 kDa, 1:2500, mouse monoclonal; BD Biosciences, Franklin Lakes, NJ, USA) and actin (42 kDa, 1:5000, mouse monoclonal; Abcam). The specific protein was detected by enhanced chemiluminescence (ECL), horseradish peroxidase (HRP) developing agents (AbFrontier, Anyang, Korea) and autoradiography film (GE Healthcare, Amersham, Bucks, UK), while band quantitation was performed with Geliance 600 (PerkinElmer, Waltham, MA, USA).

## Results

### Absorbance values of AGS gastric cancer cell lines increases within a certain range of TGF-β1 concentrations in a cell proliferation assay

The present study sought to determine changes in absorbance values due to changes in TGF-β1 concentration by performing a cell proliferation assay on the AGS cancer cell line. The study aimed to detect patterns in the absorbance value changes that could help identify the effect of increasing TGF-β1 concentration on the number of cancer cells remaining following TGF-β1 treatment. Since calculated absorbance values are based on the amount of light that remains after being absorbed by cancer cells, the values are good indicators of the number of remaining cancer cells following TGF-β1 treatment. The results are shown in Table I. The untreated (0 ng/ml TGF-β1) AGS cancer cell line displayed an absorbance value of 0.698, which is the average value obtained from two successive trials. The AGS cancer cell line treated with 0.5 ng/ml TGF-β1 displayed a higher absorbance value of 0.724. Cells treated with 5 ng/ml TGF-β1 had an absorbance value of 0.980, demonstrating an increase from the previous concentration. The AGS cancer cell line treated with 25 and 50 ng/ml TGF-β1 displayed lower absorbance values, thereby discontinuing the pattern exhibited by the cell proliferation assay. From this result, TGF-β1 may potentially regulate gastric cancer metastasis within the concentration range of 0–5 ng/ml.

### TGF-β1 exerts an antitumor effect on AGS cancer cell lines through cdk4 and p27 pathways

**Figure 1.**
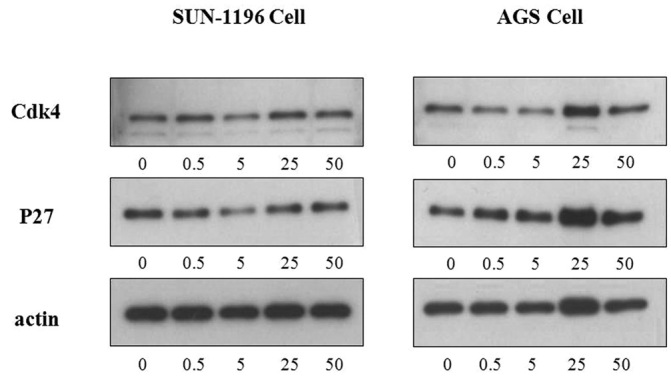
Film detection of protein bands of antibodies by western blot analysis.

**Figure 2.**
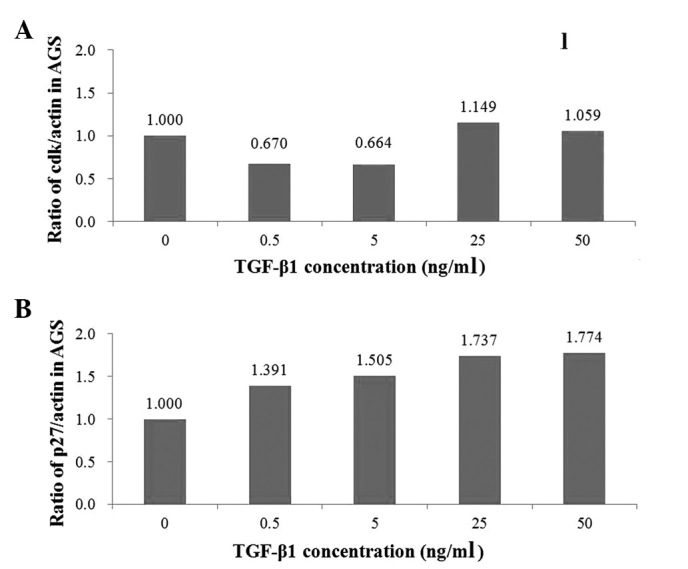
(A) Change in the quantity of cdk with increasing TGF-β1 concentration in AGS cells. (B) Change in the quantity of p27 with increasing TGF-β1 concentration in AGS cells. TGF-β1, transforming growth factor-β.

Following the cell proliferation assay, western blot analysis was performed to track the specific pathways through which TGF-β1 exerts its anti-proliferative effect on AGS cancer cell lines. Three antibodies (cdk4, p27 and actin) were subjected to western blot analysis. Their respective protein bands in each TGF-β1 concentration are reproduced in Fig. 1. From Fig. 1, a generally decreasing thickness pattern is evident for cdk4 protein bands, whereas an increasing thickness pattern is demonstrated in p27 protein bands within a certain range. This result is similar to that of the cell proliferation assay performed on AGS cancer cell lines. To ensure uniformity of the western blot analysis, another antibody (actin) was used. The actin protein band thickness indicated whether the values obtained for other antibodies were reliable, as the first three actin bands have consistent thickness for AGS cancer cell lines. From the pattern exhibited by protein bands alone, TGF-β1 appears to function through two pathways. In Fig. 2A, cdk4 values decreased from 1.000 to 0.670 and then to 0.664, with increasing TGF-β1 concentrations of 0, 0.5 and 5 ng/ml, respectively. From 25 ng/ml TGF-β1, cdk4 values began to increase. In contrast with cdk4, which decreased with increasing TGF-β1 concentrations, p27 increased from 1.000 to 1.391 and then to 1.505 with increasing TGF-β1 concentrations of 0, 0.5 and 5 ng/ml (Fig. 2B). Also dissimilar to cdk4, p27 values demonstrated consistent increases, including at 25 and 50 ng/ml of TGF-β1, where p27 values were 1.737 and 1.774, respectively.

### Absorbance values of SUN-1196 cholangiocarcinoma cell lines increase within a certain range of TGF-β1 concentrations in the cell proliferation assay

Absorbance values of SUN-1196 cholangiocarcinoma cell lines were measured with a cell proliferation assay. The change in absorbance values displayed increasing and decreasing patterns within certain ranges of TGF-β1 concentrations. As shown in Table II, SUN-1196 cancer cell lines treated with 0, 0.5 and 5 ng/ml TGF-β1 displayed a decreasing pattern of absorbance values; 0.643, 0.613 and 0.609, respectively. Cells treated with 25 and 50 ng/ml TGF-β1 displayed an increasing pattern of absorbance values; 0.626 and 0.697, respectively. This result reveals a potential antineoplastic effect of TGF-β1 on cholangiocarcinoma cells within a 5–50 ng/ml concentration range.

### TGF-β1 exerts an antitumor effect on SUN-1196 cholangiocarcinoma cancer cell lines through the p27 (and not the cdk4) pathway

**Figure 3.**
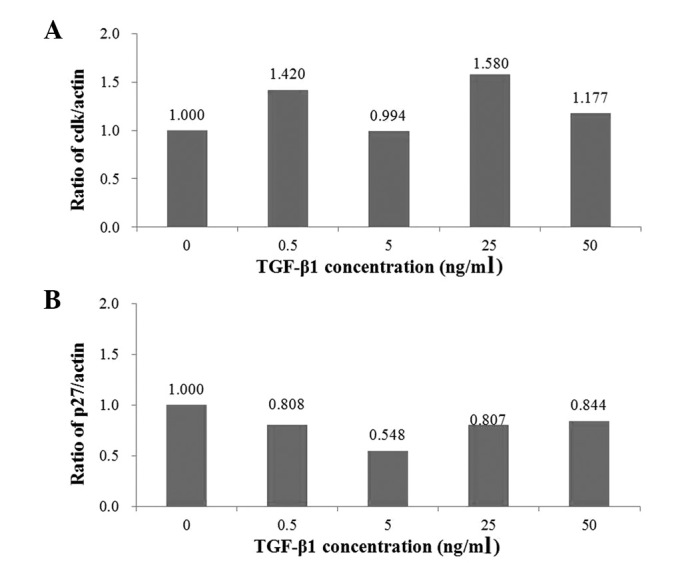
(A) Change in the quantity of cdk4 with increasing TGF-β1 concentration in SUN-1196 cells. (B) Changes in the quantity of p27 with increasing TGF-β1 concentration in SUN-1196 cells. TGF-β1, transforming growth factor-β.

The influence of TGF-β1 on SUN-1196 cholangiocarcinoma cancer cell lines was observed by western blot analysis of three antibodies; cdk4, p27 and actin. As is evident in Fig. 1, cdk4 protein bands had indiscriminate thicknesses with increasing TGF-β1 concentrations, while p27 protein bands varied in thickness according to a pattern within certain TGF-β1 concentration ranges. The actin protein bands had uniform thickness, indicating that the results for other antibody protein bands are reliable. As shown in Fig. 3A, cdk4 values (1.000, 1.420, 0.994, 1.580 and 1.177) varied without any distinct patterns. However, Fig. 3B reveals that p27 values decreased from 1.000 to 0.808 and then to 0.548 with increasing TGF-β1 concentrations of 0, 0.5 and 5 ng/ml. The p27 values also increased from 0.548 to 0.807 and then to 0.844 with increasing TGF-β1 concentrations of 5, 25 and 50 ng/ml.

## Discussion

Within a certain concentration range, TGF-β1 was revealed to play an antitumor role in two types of cancer; gastric cancer and cholangiocarcinoma. According to the cell proliferation assay results for AGS cancer cell lines, the absorbance values of AGS cells treated with 0, 0.5 and 5 ng/ml TGF-β1 consistently increased. The pattern of increasing absorbance indicates that the number of AGS cells decreased with increasing TGF-β1 concentration, as absorbance is measured by detecting the amount of light remaining after being partially absorbed by cells. A higher absorbance value indicates that the machine detected a greater amount of light and the cells absorbed less light, demonstrating that fewer cells are present. Since the absorbance value was highest in AGS cells treated with 5 ng/ml TGF-β1, the smallest number of AGS cells remained in that concentration. This result demonstrated the antitumor role of TGF-β1 within a specific concentration range. In short, TGF-β1 plays an antitumor role in gastric cancer cell lines when its concentration is between 0 and 5 ng/ml. The western blot analysis results confirmed the aforementioned analysis of the cell proliferation assay and explained the specific pathways through which TGF-β1 exerts an anti-neoplastic effect on gastric cancer cells. The unitless cdk4 and p27 in Fig. 2A and B indicate the quantity of each antibody detected by film detection technique in the western blot analysis. As shown in Fig. 2A, the quantity of cdk4 decreased with an increasing TGF-β1 concentration between 0 and 5 ng/ml. As shown in Fig. 2B, the quantity of p27 increased with increasing TGF-β1 concentration between 0 and 50 ng/ml. These two results confirmed our hypothesis that TGF-β1 exerts an antitumor effect on gastric cancer cell lines through the downregulation of cdks (cdk4) and the upregulation of p27. The western blot analysis results were also concordant with the cell proliferation assay results, which suggested that TGF-β1 plays an antitumor role in AGS when its concentration is between 0 and 5 ng/ml. The present study demonstrated that TGF-β1 exerts an antitumor effect on gastric cancer cells through two pathways, cdk4 and p27, by downregulating cdk4 and by upregulating p27. Bhayal *et al* revealed that TGF-β1 may be a risk factor of genetic susceptibility to gastric cancer in the south Indian population (23). Yuan *et al* demonstrated that TGF-β1 plays an antitumor role in gastric cancer rather than inducing gastric cancer cells to escape human immunological surveillance (24–26). Our results are concordant with those of Yuan *et al*(24).

As a result of the SUN-1196 cell proliferation assay, another distinct pattern of increasing absorbance data was identified within the TGF-β1 concentration range of 5 to 50 ng/ml, which is different from the range found in AGS gastric cancer cell lines. A lower absorbance value indicates that the machine detected less light, thus the SUN-1196 cells absorbed more light. A greater number of SUN-1196 cells remained when retreated with 5 ng/ml TGF-β1, and this number decreased with increasing TGF-β1 concentrations of 5, 25 and 50 ng/ml, as indicated in Table II. These results suggest that TGF-β1 has an antitumor and anti-proliferative effect on cholangiocarcinoma cells when its concentration ranges from 5 to 50 ng/ml. Fig. 3A and B reaffirmed the anti-neoplastic influence of TGF-β1 on cholangiocarcinoma cells and revealed the pathway through which the antitumor effect is exerted. As with the AGS cell western blot analysis results, the cdk4 and p27 values in Fig. 3 refer to the quantity of antibodies indicated by the thickness of protein bands in Fig. 1. Due the lack of a pattern in the distribution of cdk4 values with increasing TGF-β1 concentration (Fig. 3A), we concluded that TGF-β1 does not follow the cdk4 pathway and so does not downregulate cdk4 as was hypothesized previously. However, Fig. 3B revealed a clear pattern of increase in the quantity of p27 within a range of 5–50 ng/ml. This pattern confirmed that TGF-β1 exerts an anti-cancer influence via the p27 pathway as was hypothesized. This result was also concordant with the cell proliferation assay, which suggested that TGF-β1 is at its most effective as an anti-cancer agent at the concentration range of 5–50 ng/ml. In brief, TGF-β1 has an antitumor effect on cholangiocarcinoma cells through the p27 pathway, but not through the cdk4 pathway. Zen *et al* demonstrated that TGF-β1 did not influence the cell-proliferative activities of three cultured human intrahepatic cholangiocarcinoma (ICC) cells (27). However, the present study revealed that when its concentration is between 5 and 50 ng/ml, TGF-β1 has an anti-proliferative effect on cholangiocarcinoma cells. Zen *et al* also demonstrated that cyclin D1 is key for ICC cells attaining TGF-B1 resistance. However, the present study has demonstrated (by western blot analysis) that through upregulation of p27, TGF-β1 is capable of deterring cholangiocarcinoma proliferation, thus nullifying the resistance of cholangiocarcinoma to TGF-β1 (27). Furthermore, Shimizu *et al* revealed that TGF-β1 stimulation in ICC results in cellular proliferation. The present study demonstrated that TGF-β1 stimulation in cholangiocarcinoma resulted in downregulation of cellular proliferation when TGF-β1 concentration was between 5 and 50 ng/ml (28).

In conclusion, certain concentrations of TGF-β1 play antitumor roles in gastric cancer through the downregulation of cdk4 and the upregulation of p27. These TGF-β1 concentrations also have antitumor roles for cholangiocarcinoma through the upregulation of p27. These results bring us a step closer to finding a cure for cholangiocarcinoma and gastric cancer. In future studies, we intend to increase the number of antibodies for western blot analysis, the types of cancer cell being tested and the concentrations of TGF-β1. Additionally, PCR will be included to refine our conclusions. With these additions, we may be able to produce more significant results that may further enhance the effort to find a novel cure for cancers.

## Figures and Tables

**Table I.** Cell proliferation assay for AGS cells.

	AGS absorbance			
TGF-β1 concentration (ng/ml)	1st trial	2nd trial	Average	Standard deviation
0	0.697	0.699	0.698	0.001
0.5	0.714	0.734	0.724	0.014
5.0	0.911	1.049	0.98	0.098
25	0.7	0.834	0.767	0.095
50	0.706	0.728	0.717	0.016

TGF-β1, transforming growth factor-β; AGS, human gastric cancer cell line.

**Table II.** Cell proliferation assay for SUN-1196 cells.

	SUN-1196 absorbance			
TGF-β1 concentration (ng/ml)	1st trial	2nd trial	Average	Standard deviation
0	0.645	0.641	0.643	0.003
0.5	0.611	0.615	0.613	0.003
5	0.604	0.613	0.609	0.006
25	0.617	0.635	0.626	0.013
50	0.675	0.718	0.697	0.030

TGF-β1, transforming growth factor-β1; SUN-1196, human cholangiocarcinoma cell line.
